# Authors’ Response

**DOI:** 10.4269/ajtmh.19-0406b

**Published:** 2019-10

**Authors:** Ana Lineth Garcia, Richard Reithinger

**Affiliations:** CUMETROP, Universidad Mayor de San Simón, Cochabamba, Bolivia; RTI International, Washington, District of Columbia

Dear Sir,

We thank Mollinedo et al.^1^ for their interest in and comments on our 2009 review of the leishmaniases in Bolivia.^2^

Indeed, Mollinedo et al.^1^ are correct: in one instance within our article, we unfortunately and incorrectly assigned Bolivia’s sole visceral leishmaniasis (VL) focus in the Yungas region to the Department of Beni. We doubt, however, that this error “continues to confuse researchers” because 1) our article correctly states elsewhere that “all VL cases are reported from the Yungas region in the La Paz department” [p. 705] and “*Leishmania* (*L.*) *infantum*, the causative agent of VL, was isolated from or detected in patients, dogs, and the insect vector *Lutzomyia longipalpis* in the Yungas region in the Department of La Paz.” [p. 706] and 2) cross-checking the original report by Desjeux P et al.,^3^ as well as a simple Google search would have clarified the correct location of the Yungas VL focus.

As pointed out by Mollinedo et al.,^1^ some of the data and program information presented in our review came from normative documents by the Ministry of Health.^4,5^ Given that these documents did not have a suggested citation and were in addition endorsed by several Ministry of Health leaders besides having Mollinedo et al. as document contributors, we opted to assign authorship of the document to “anonymous” rather than “Mollinedo et al.” or “Ministry of Health”—which is not an unusual practice when referencing official Ministry of Health strategy, policy, and other normative guidance documents.

Mollinedo et al.^1^ correctly stated that between 1983 and 2006, 35,714 cutaneous leishmaniasis (CL) cases were reported. However, as per Table 2 in Ref. 3, these numbers include “all forms of leishmaniasis”; as stated in our article, this includes “31,095 cases of localized CL (LCL) and 4,619 cases of mucosal leishmaniasis” [p.704], that is 35,714 CL cases—this was based on the information we obtained when conducting our review in 2008.

While we acknowledge that Figures 1B and 2 did not refer to Mollinedo et al.^3^ in the figure legends per se, the article’s narrative introducing the figures and the data presented therein makes ample reference to Mollinedo et al. and the National Program of Leishmaniasis Control. Also, we note that Figure 2 specifically refers to LCL rather than all leishmaniasis cases (see previous paragraphs) and, again, was based on the information available to us when conducting our review at that time.

Given that our review has come of age, we would welcome to collaborate with Mollinedo et al. to document progress in leishmaniases’ prevention and control efforts in Bolivia over the past decade.
